# Antiviral innate immune response in non-myeloid cells is augmented by chloride ions via an increase in intracellular hypochlorous acid levels

**DOI:** 10.1038/s41598-018-31936-y

**Published:** 2018-09-11

**Authors:** Sandeep Ramalingam, Baiyi Cai, Junsheng Wong, Matthew Twomey, Rui Chen, Rebecca M. Fu, Toby Boote, Hugh McCaughan, Samantha J. Griffiths, Jürgen G. Haas

**Affiliations:** 1 Department of Laboratory Medicine, NHS Lothian, Edinburgh Royal Infirmary, Edinburgh, UK; 2 Division of Infection and Pathway Medicine, University of Edinburgh, Edinburgh, UK

## Abstract

Phagocytes destroy ingested microbes by producing hypochlorous acid (HOCl) from chloride ions (Cl^−^) and hydrogen peroxide within phagolysosomes, using the enzyme myeloperoxidase. HOCl, the active ingredient in bleach, has antibacterial/antiviral properties. As myeloperoxidase is needed for HOCl production, non-myeloid cells are considered incapable of producing HOCl. Here, we show that epithelial, fibroblast and hepatic cells have enhanced antiviral activity in the presence of increasing concentrations of sodium chloride (NaCl). Replication of enveloped/non-enveloped, DNA (herpes simplex virus-1, murine gammaherpesvirus 68) and RNA (respiratory syncytial virus, influenza A virus, human coronavirus 229E, coxsackievirus B3) viruses are inhibited in a dose-dependent manner. Whilst treatment with sodium channel inhibitors did not prevent NaCl-mediated virus inhibition, a chloride channel inhibitor reversed inhibition by NaCl, suggesting intracellular chloride is required for antiviral activity. Inhibition is also reversed in the presence of 4-aminobenzoic hydrazide, a myeloperoxidase inhibitor, suggesting epithelial cells have a peroxidase to convert Cl^−^ to HOCl. A significant increase in intracellular HOCl production is seen early in infection. These data suggest that non-myeloid cells possess an innate antiviral mechanism dependent on the availability of Cl^−^ to produce HOCl. Antiviral activity against a broad range of viral infections can be augmented by increasing availability of NaCl.

## Introduction

Chloride, the most abundant anion in humans, is an important prerequisite for the innate immune response mediated by phagocytes and neutrophils^1^. Resting neutrophils have a four- to five-fold higher intracellular Cl^−^ concentration than expected for passive transfer^2^. Chloride transport across hydrophobic lipid membranes requires protein carriers such as chloride channels, anion-chloride exchangers or cation-chloride co-transporters^3^. Within phagosomes, myeloperoxidase (MPO) mediates the conversion of Cl^−^ and hydrogen peroxide (H_2_O_2_) to hypochlorous acid (HOCl)^1^. Both H_2_O_2_ and HOCl have antimicrobicidal activity, however HOCl is much more potent^4^. An activated neutrophil is estimated to produce 1.6 × 10^6^ molecules of HOCl per second^1^. Within phagosomes, an estimated 28–72% of the oxygen consumed is converted into HOCl^5^. Hence a continuous supply of chloride is required for HOCl generation^5^.

In cystic fibrosis, the mutation in cystic fibrosis transmembrane conductance regulator (CFTR), (a cAMP/PKA-activated Cl^−^ channel) leads to decreased chlorination and killing of ingested bacteria^1^. The uptake of chloride ions is lower in nasal epithelial cells of individuals with cystic fibrosis compared to cells from individuals without cystic fibrosis^6^.

In the 1960’s, Speir R.W. reported the possible antiviral activity of chloride/halide salts^7^. Exposure of mengovirus (a Cardiovirus, Picornaviridae family) to 150 mMol NaCl (37 °C for 2 hours) led to a 4 log_10_ reduction in LD_50_^7^. A significant drop in LD_50_ was also seen with other chloride salts [KCl (150 mMol); MgCl_2_/CaCl_2_ (75 mMol)] and halide salts (150mMol NaBr/NaI)^7^. Here we report that both DNA and RNA viruses, enveloped and non-enveloped viruses, cultured in non-myeloid cells are inhibited in the presence of NaCl. Our data suggests that viral inhibition is an intracellular process and not a direct effect of NaCl on the virus particles or on viral adsorption. Viral inhibition is reversed when chloride ions (but not sodium ions) are prevented from entering the cell. Viral inhibition is associated with an increase in the production of intracellular hypochlorous acid (HOCl). This is corroborated by the reversal of viral inhibition in the presence of a known myeloperoxidase inhibitor. Hence, HOCl production is an innate antiviral mechanism which works against DNA, RNA, enveloped and non-enveloped viruses.

## Results

**Figure 1:**
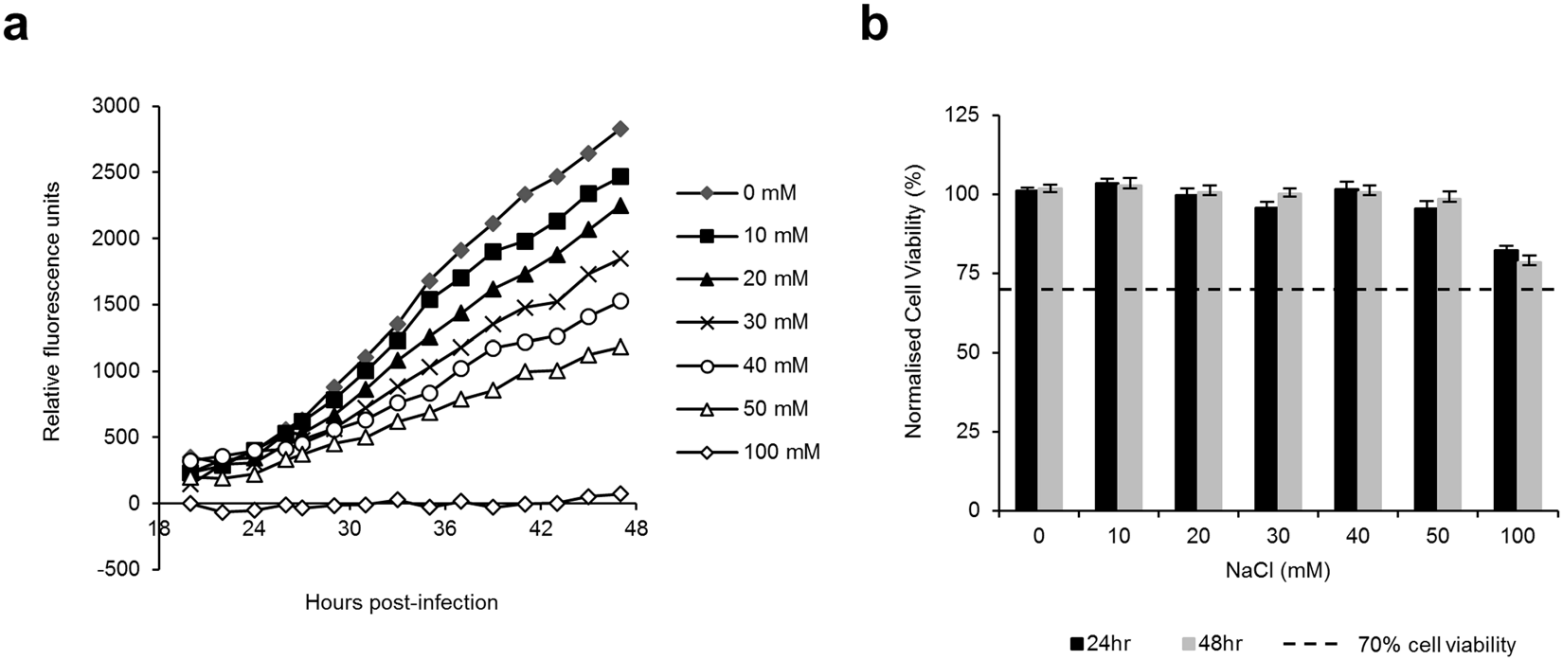
Dose dependent inhibition of HSV-1 by sodium chloride: (**a**) Time course analysis of HSV-1 in the presence of NaCl: HeLa cells were infected with HSV-1-GFP (MOI 0.5) for 1 hour before the inoculum was removed and replaced with increasing concentrations of NaCl in medium (in triplicate). NaCl (mM) values are over and above that found in DMEM (110 mM). Virus replication was monitored as a function of GFP fluorescence over time. (**b**) Viability of HeLa cells is not significantly impaired in the presence of NaCl. HeLa cells were treated with increasing concentrations of NaCl (in triplicate). 24 and 48 hours post-treatment cellular viability was determined with CellTiter-Blue (Promega) by the ability of cells to metabolise the substrate to produce a fluorescent end-product. Cell viability was normalised to untreated cells (0 mM NaCl). Viability below 70% is evidence of cytotoxicity. Error bars indicate the standard error of the mean of 3 independent experiments carried out in triplicate.

**Figure 2:**
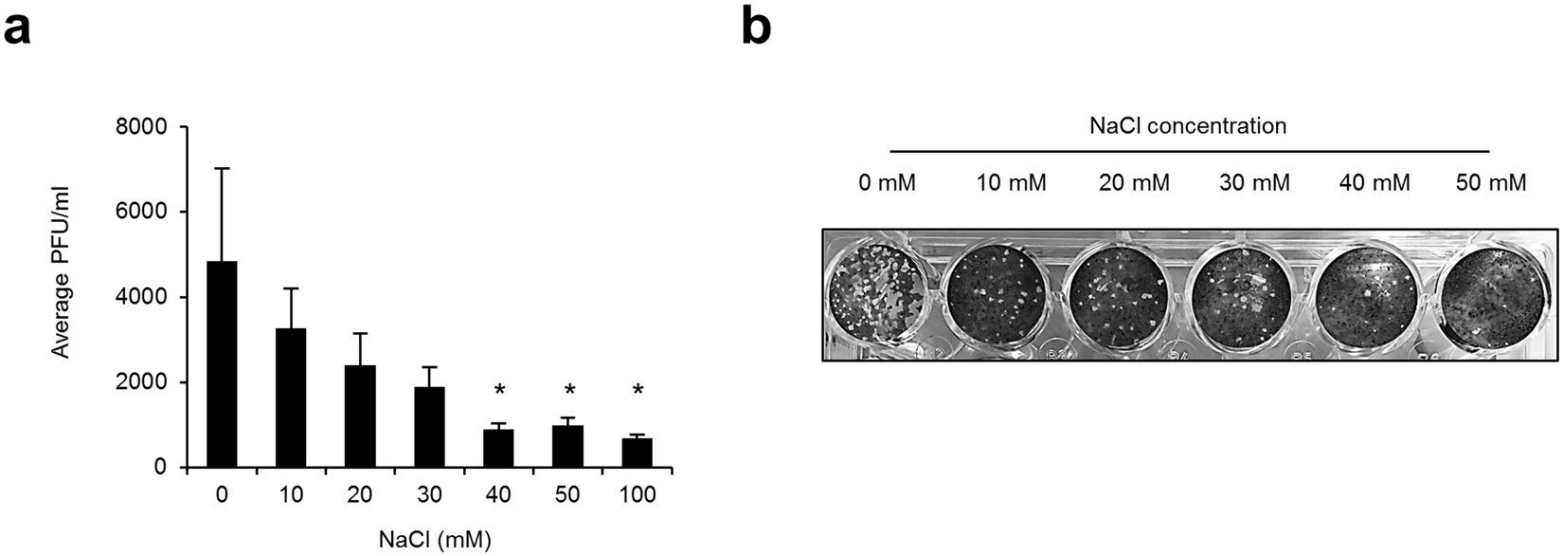
Confirmation of inhibition of HSV-1 by plaque forming assay: (**a**) HSV-1 virion release is inhibited by NaCl: HeLa cells were infected with HSV-1 (MOI 0.5) for 1 hour before inoculum was removed and replaced with increasing concentrations of NaCl. Supernatant was harvested after 48 hours and viral titer quantified by plaque assay on Vero cell monolayers, in the absence of NaCl, as plaque forming units (PFU) per ml. Error bars represent the standard error of the mean of three experiments carried out in duplicates. *p < 0.05 when compared to 0 mM NaCl (**b**) A representative image of NaCl inhibition of HSV-1 by plaque forming assay. NaCl (mM) values are over and above that found in DMEM (110 mM).

To test whether NaCl has an inhibitory effect on viruses, we first conducted inhibition experiments with herpes simplex virus-1 (HSV-1). A HSV-1 reporter virus expressing enhanced green fluorescence protein (eGFP) was tested in HeLa cells (cervical epithelial cells) in the presence of increasing concentrations of NaCl, and fluorescence intensity was measured at regular intervals over 48 hours. A dose-dependent reduction of HSV-1 replication was observed for NaCl concentrations up to 100 mM (Fig. 1a). The NaCl concentrations shown are concentrations additional to the NaCl already present in media (110 mM) and not final concentrations. Cell viability was >70% at all concentrations tested, suggesting that the observed inhibitory effect was not caused by cytotoxicity (Fig. 1b). To address the possibility of GFP fluorescence being inhibited in the presence of NaCl or by a metabolite, HSV-1 virus production was also measured by plaque forming assays. A clear dose-dependent reduction in plaque forming units was detected (Fig. 2a,b). This confirms the viral inhibition seen in the GFP fluorescence assay is genuine.

**Figure 3:**
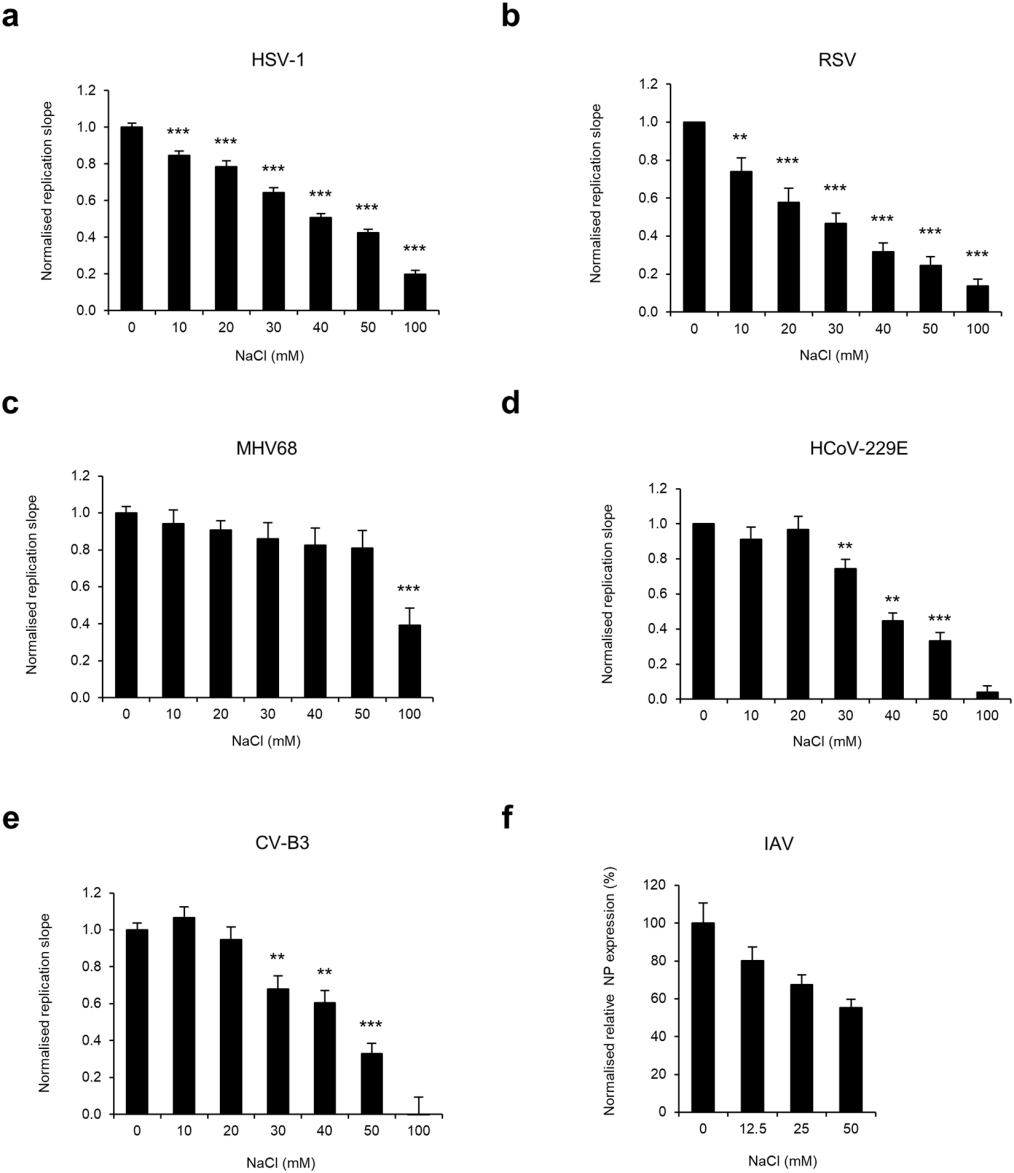
DNA and RNA viruses are inhibited by sodium chloride: Dose-dependent inhibition of (**a**) HSV-1, (**b**) RSV, (**c**) MHV68, (**d**) HCoV-229E, (**e**) CV-B3 and (**f**) IAV by NaCl. Cells were infected with virus for 1 hour before virus was replaced with media containing increasing concentrations of NaCl. Viral replication was quantified by measuring eGFP fluorescence intensity over multiple rounds of replication and determining the slopes of the growth curves in time course analyses, with the exception of IAV, which was quantified by qRT-PCR for the viral nucleoprotein (NP). With the exception of IAV, which represents one experiment of duplicates, all error bars represent the standard error of the mean of at least two independent experiments carried out in triplicate. *p < 0.05; **p < 0.01 and ***p < 0.001 when compared to 0 mM NaCl. NaCl (mM) values are over and above that found in DMEM (110 mM).

**Figure 4:**
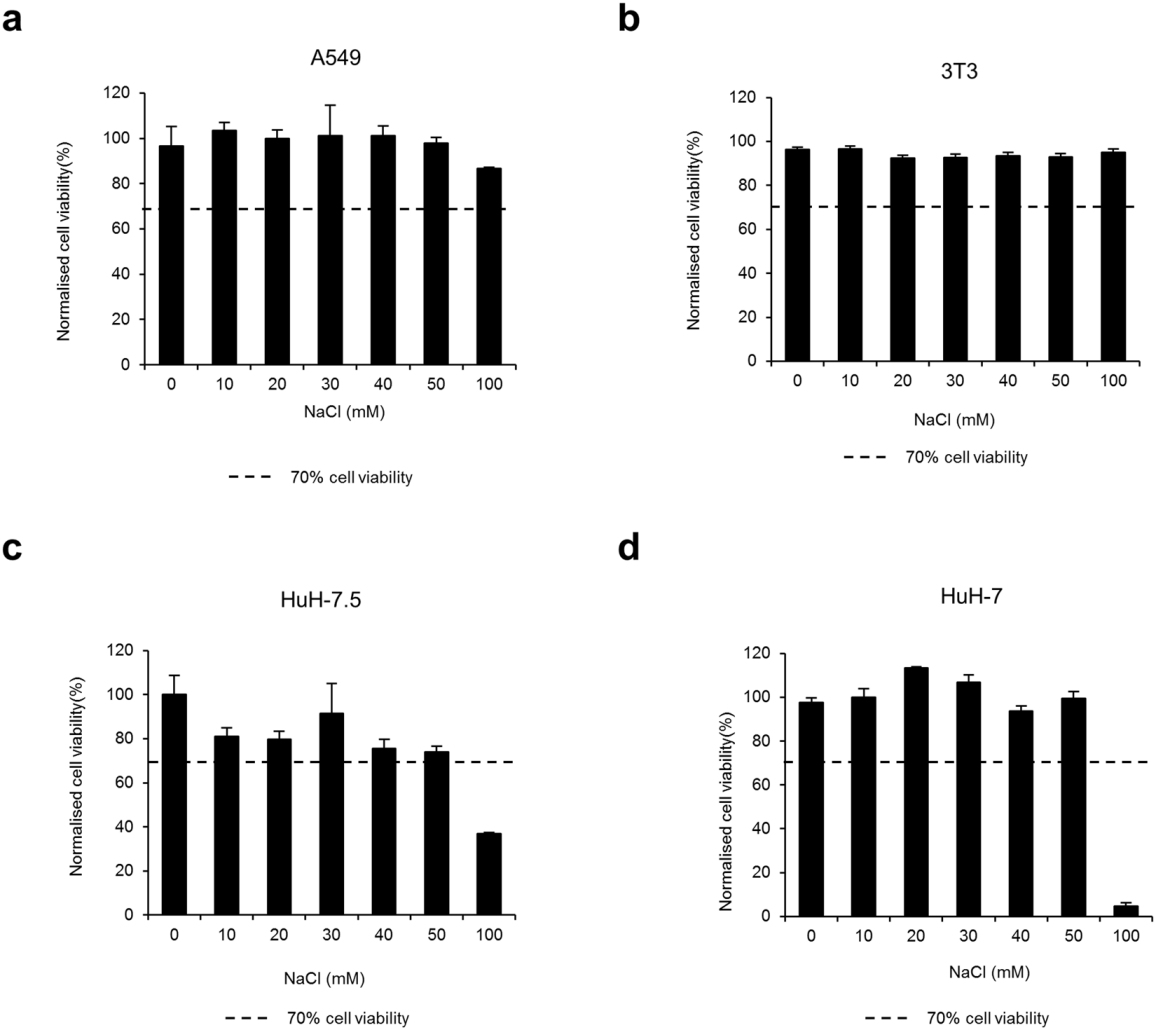
Viability of different non-myeloid cells in the presence of sodium chloride: Viability of A549 (**a**), 3T3 (**b**), HuH-7.5 (**c**), and HuH-7 (**d**) cells were not significantly impaired in the presence of NaCl. Cells were treated with increasing concentrations of NaCl (in triplicate). 48 hours post-treatment cellular viability was determined with CellTiter-Blue (Promega) by the ability of cells to metabolise the substrate to produce a fluorescent end-product. Cell viability was normalised to untreated cells (0 mM NaCl). Viability below 70% is evidence of cytotoxicity. While A549 and 3T3 cells were viable up to 100 mM NaCl, HuH-7.5 and HuH-7 cells were viable only up to 50 mM NaCl. Error bars represent the standard error of the mean of 3 independent experiments carried out in triplicate. NaCl (mM) values are over and above that found in DMEM (110 mM).

To test whether other viruses (DNA, RNA, enveloped and non-enveloped) are inhibited as well, viral inhibition experiments were conducted with eGFP labelled murine gammaherpesvirus 68 (MHV68; an enveloped, DNA gammaherpesvirus) in 3T3 fibroblast cells, respiratory syncytial virus (RSV; an enveloped RNA pneumovirus) in HeLa cells, coxsackievirus B3 (CV-B3; a non-enveloped picornavirus) in HuH-7.5 hepatoma cells, human coronavirus 229E (HCoV-229E – an enveloped RNA coronavirus) in HuH-7 hepatoma cells and un-labelled influenza A virus (IAV; an enveloped RNA orthomyxovirus; strain Udorn) in A549 respiratory epithelial cells. A reduction in viral replication is seen in the presence of increasing concentrations NaCl for all viruses tested (Fig. 3a–f). The viability of A549, 3T3, and HuH-7.5 and HuH-7 cells are shown in Fig. 4. There was a significant dose-dependent reduction of viral replication for HSV-1 (Fig. 3a, 80% reduction, p < 0.00001) and RSV (Fig. 3b, 86% reduction, p = 0.00001) seen at 100 mM NaCl over and above the concentration of NaCl in DMEM (110 mM). This concentration of NaCl is not cytotoxic to HeLa cells (Fig. 1b). Furthermore, there was a significant reduction in replication of both HSV-1 and RSV in the presence of as little as 10 mM NaCl over and above the concentration of NaCl in DMEM. The pattern of inhibition by NaCl appears to be dependent on the virus and/or the cell type. MHV68 replication (3T3 cells) was only significantly inhibited at 100 mM of NaCl over and above the concentration in DMEM (Fig. 3c, p = 0.00006), a concentration that was not cytotoxic to 3T3 cells (Fig. 4b). HCoV-229E and CV-B3 were both significantly inhibited from 30–50 mM NaCl over and above the concentration in DMEM (p < 0.01; Fig. 3d and e). However, as cell viability was very low at 100 mM NaCl in both HuH7 (HCoV-229E) and HuH7.5 (CV-B3) cells (5% and 37%, respectively; Fig. 4c and d), inhibition of these viruses by NaCl should only be considered up to 50 mM NaCl. Finally, a dose-dependent reduction in viral replication was also seen for influenza A virus in A549 cells (Fig. 3f). P-values were not calculated for influenza A virus as the experiment was only done once.

**Figure 5:**
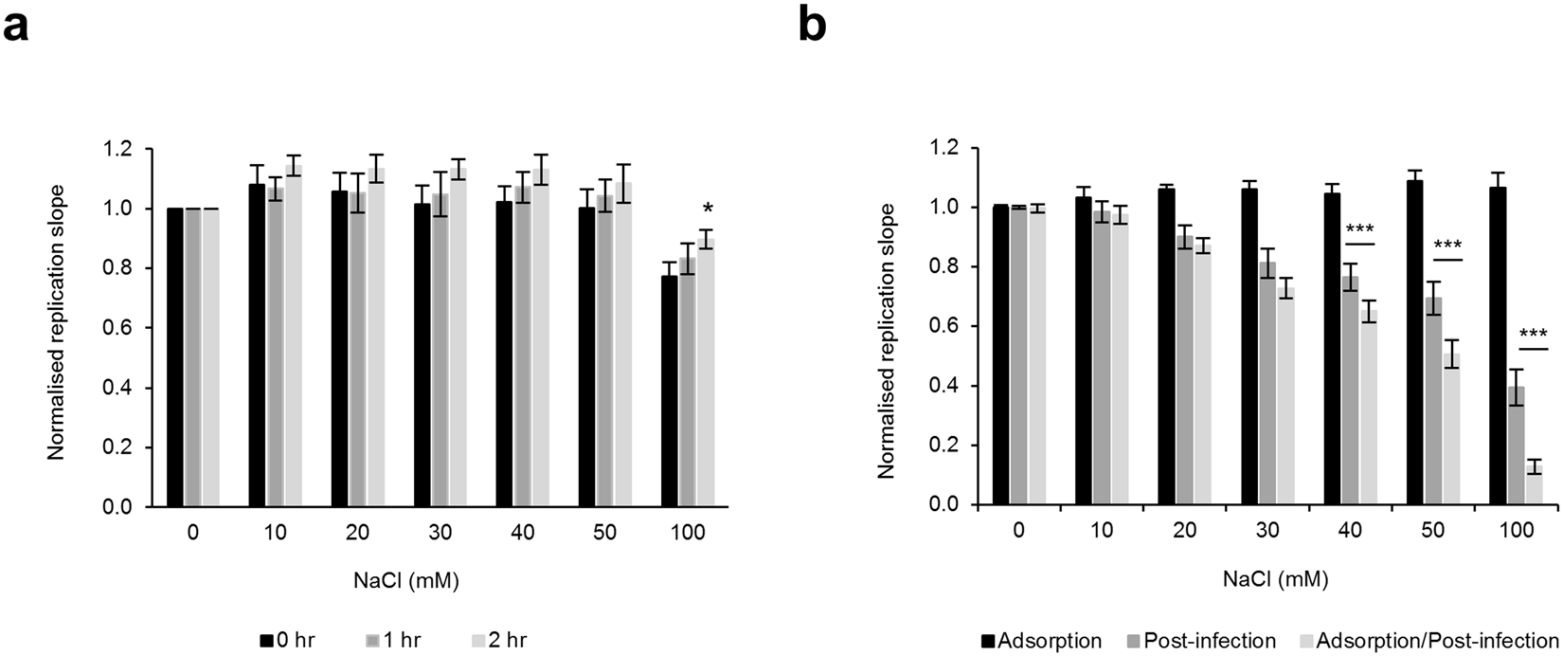
Sodium chloride inhibits HSV-1 infection of HeLa cells at a stage post-entry: (**a**) eGFP HSV-1 was pre-incubated with increasing concentrations of NaCl for 0, 1 or 2 hours before adsorption to HeLa cells (MOI 0.5). After adsorption, inoculum was replaced with media. Virus replication was monitored as a function of fluorescence over time. Error bars represent the standard error of the mean of three biological replicates. *p < 0.05 compared to 0 hours at the corresponding concentration of NaCl (**b**) HeLa cells were infected with eGFP HSV-1 (MOI 0.5) and treated with increasing concentrations of NaCl at different stages of infection: Adsorption (present during the 1 hour of adsorption alone), Post-infection (NaCl added following removal of inoculum), or Adsorption/Post-Infection (NaCl present both during adsorption and following removal of inoculum). Error bars represent the standard error of the mean of three independent experiments carried out in triplicate. ***p < 0.001 when compared to 0 mM NaCl. NaCl (mM) values are over and above that found in DMEM (110 mM).

Subsequently we used eGFP HSV-1 to identify the mechanism of inhibition. To determine if viral inhibition was a direct effect of NaCl on the virus, eGFP HSV-1 was pre-incubated with media (no added NaCl) or increasing concentrations of NaCl (10–100 mM) for 0, 1 and 2 hours before virus was adsorbed. Pre-exposure to NaCl did not affect viral replication at any concentration of NaCl (Fig. 5a). After 2 hours pre-exposure there was a suggestion of increased replication, although this was minimal. Compared to other concentrations, there was a small (10–25%) reduction in viral replication at 100 mM NaCl, probably due to passive transfer of NaCl.

Next, we determined if viral inhibition happened at the stage of viral adsorption, or happened intracellularly, after adsorption. Cells were exposed to media alone or increasing concentrations of NaCl during virus adsorption alone, after virus adsorption (i.e. during virus replication alone) or both during viral adsorption and replication. For each of these conditions, replication slopes were normalized to control cells exposed to media alone. When the cells were exposed to NaCl only during adsorption with eGFP-HSV-1 there was no reduction in viral replication with increasing NaCl concentrations (Fig. 5b). However, significant inhibition of viral replication was seen when as little as 20 mM NaCl was available during virus replication alone (Fig. 5b, p = 0.02) or both during adsorption and replication (Fig. 5b, p = 0.0006). These data suggest that virus inhibition in the presence of NaCl is an intracellular mechanism.

**Figure 6:**
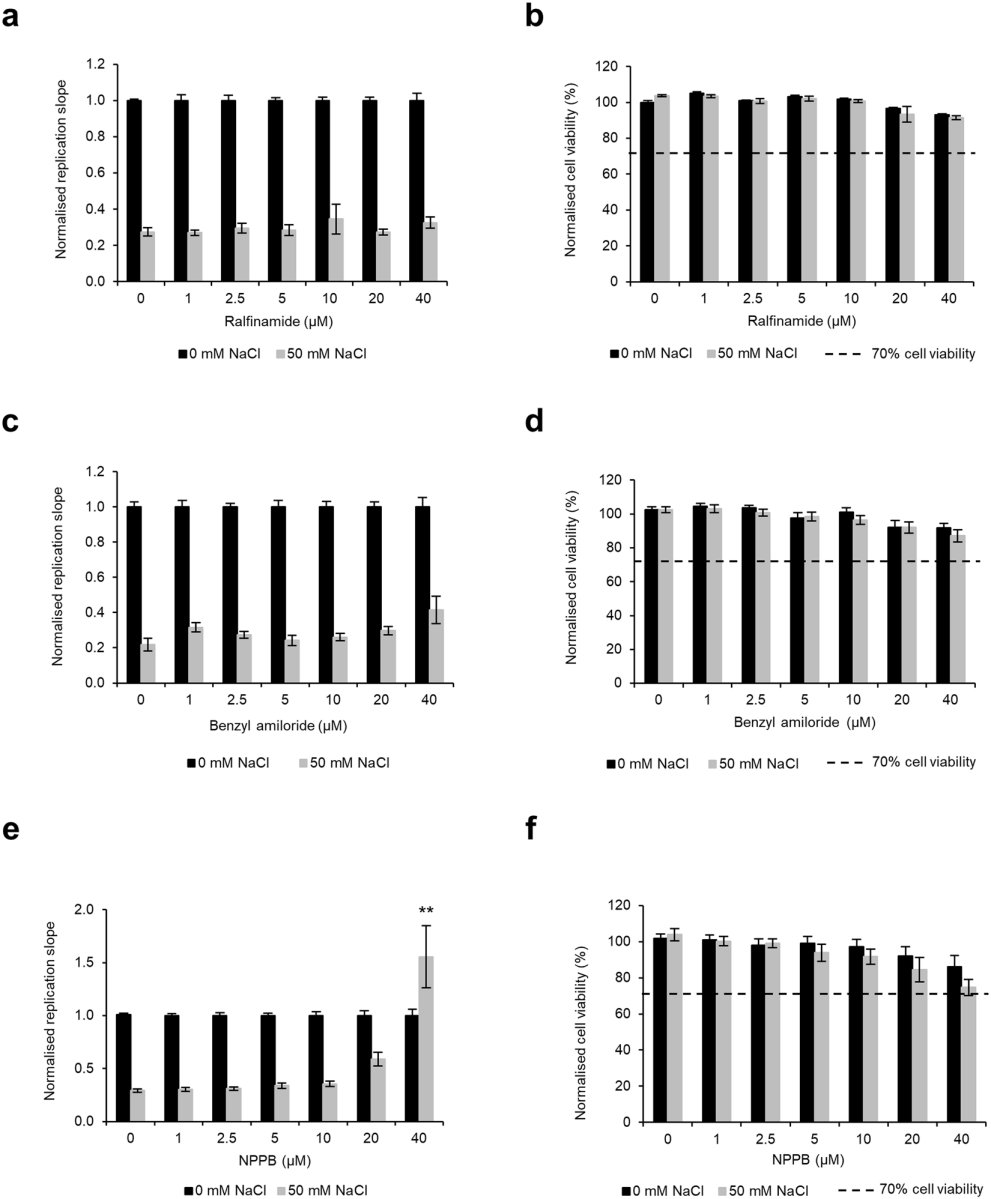
Inhibition of Cl^−^ but not Na^+^ transport restores HSV-1 replication in the presence of NaCl: HeLa cells were treated for 24 hours with increasing concentrations of ralfinamide (**a**,**b**) a voltage gated sodium channel blocker; or benzyl amiloride **(c,d)** an epithelial sodium channel blocker or (**e,f**) 5-nitro-2-(3-phenylpropyl-amino) benzoic acid (NPPB) a chloride channel blocker and infected with HSV-1-GFP at a MOI of 0.5. After 1 hour the inoculum was removed and replaced with increasing concentrations of inhibitor and either 0 mM (blue) or 50 mM NaCl (grey). Virus replication was monitored as a function of GFP fluorescence over time and normalised to the no salt control for each channel blocker concentration. Error bars represent the standard error of the mean of multiple replicates. (**b**,**d**,**f**) HeLa cells were treated with increasing concentrations of ralfinamide (**b**), benzyl amiloride (**d**) or NPPB (**f**) with 0 mM or 50 mM NaCl. After 24 and 48 hours, cell viability was determined and normalised to untreated cells (0 mM NaCl, 0 mM inhibitor). Error bars represent the standard error of the mean of three experiments carried out in triplicate. **p < 0.01 when compared to 0 μM NPPB. NaCl (mM) values are over and above that found in DMEM (110 mM).

We further investigated which of the two ions (Na^+^ or Cl^−^) plays a role in NaCl-mediated inhibition of HSV-1 replication. For this, HeLa cells were infected with eGFP-HSV-1 in media alone or with 50 mM NaCl in the presence of increasing concentrations of ion channel blockers. It might be expected that blocking transport of an important ion may block viral inhibition by NaCl. Neither the voltage gated sodium channel inhibitor Ralfinamide nor Benzyl amiloride, an inhibitor of epithelial sodium channels, reversed inhibition of HSV-1 by 50 mM NaCl (Fig. 6a and c). We then blocked chloride channels with increasing concentrations of 5-nitro-2-(3-phenylpropyl-amino) benzoic acid (NPPB). Blocking chloride channels with NPPB led to a significant reversal of viral inhibition by 50 mM of NaCl, with 40 μM NPPB even enhancing replication beyond levels seen with media alone (60% increase in replication; p = 0.003) (Fig. 6e). As the combination of NaCl with all the inhibitors was not cytotoxic to HeLa cells (viability >70%; Fig. 6b,d and f), these data suggest the influx of Cl^−^ ions is essential for the inhibition of viral replication by NaCl.

**Figure 7:**
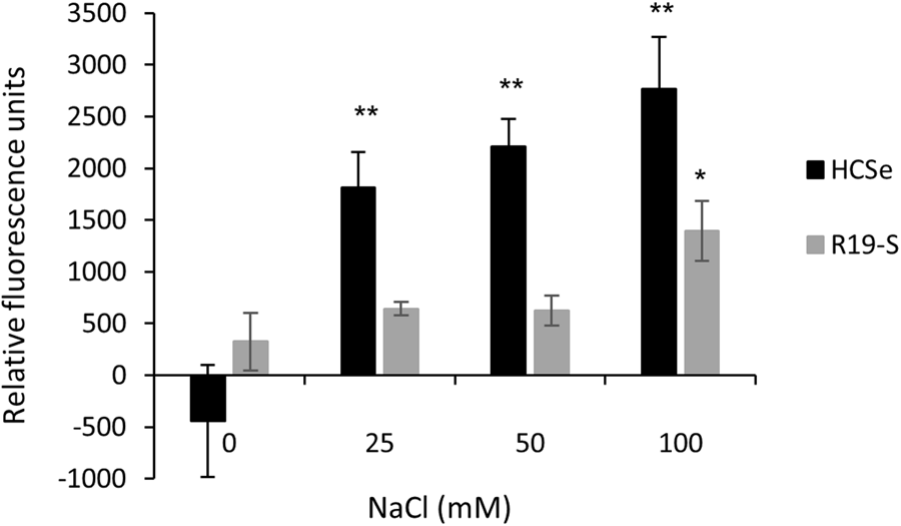
HSV-1 is inhibited in the presence of NaCl by increased production of intracellular hypochlorous acid: HeLa cells were infected with non-GFP labelled HSV-1 at MOI 0.5 for 1 hour before treating with increasing concentrations of NaCl. After 6 hours, cells were washed and stained with 10 μM HCSe^8^ (a BODIPY-based green fluorescent probe which is rapidly and specifically oxidized by HOCl to emit a fluorescence signal) or R-19S (a rhodamine fluorophore)^9^ for 30 minutes. Fluorescence was measured after cells were washed twice with PBS. Fluorescence values were normalized to uninfected cells treated with the corresponding NaCl concentration. NaCl (mM) values are over and above that found in DMEM (110 mM). Error bars represent the standard error of the mean of three biological replicates. *p < 0.05 and **p < 0.01 when compared to 0 mM NaCl.

Given the importance of chloride ion transport, there was a possibility that the inhibition of HSV-1 by NaCl was due to production of intracellular HOCl, as seen in phagocytes. Hence, we investigated if increasing concentrations of NaCl led to enhanced production of HOCl during viral infection. For this experiment, non-fluorescent HSV-1 was used. HOCl was detected with the BODIPY-based fluorescent probe hypochlorite sensor using selenium (HCSe)^8^ and a rhodamine fluorophore R19-S^9^, 6 hours post-adsorption. Whilst an increase in HOCl was seen with both dyes (relative fluorescence units ~2800 for HCSe versus ~1400 for R19-S at 100 mM NaCl), a significant increase in HOCl production in response to increasing NaCl concentrations was clearly seen with HCSe (p = 0.001 at 100 mM NaCl) and less so with R19-S (p = 0.04 at 100 mM NaCl; Fig. 7). An increase in HCSe signal with increase in concentration of NaCl was also seen as early as 2 hours post-adsorption (Data not shown).

**Figure 8:**
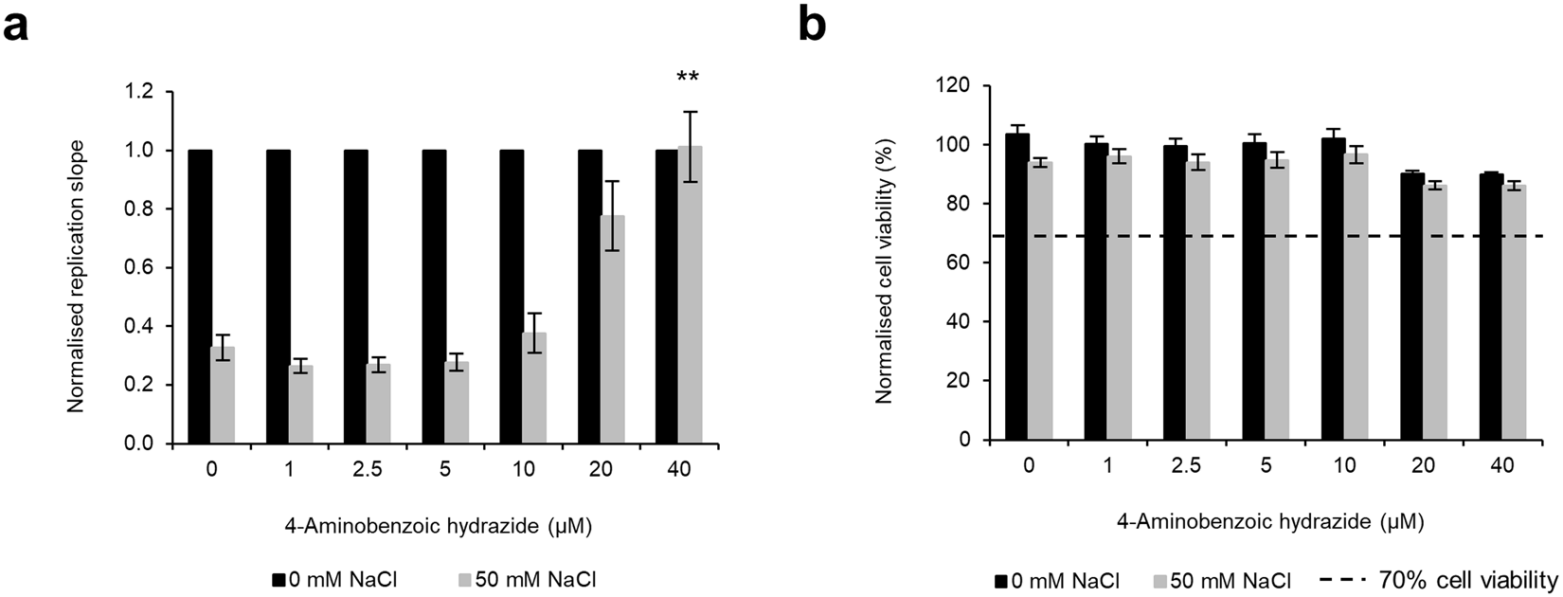
Inhibition of myeloperoxidase restores HSV-1 replication in the presence of NaCl: (**a**) Production of HOCl requires a functional peroxidase enzyme. HeLa cells were treated for 24 hours with increasing concentrations of the myeloperoxidase (MPO) inhibitor 4-Aminobenzoic hydrazide (4ABAH) before infecting with HSV-1-GFP at MOI 0.5. Following 1-hour incubation, inoculum was removed and replaced with increasing concentrations of inhibitor and either 0 or 50 mM NaCl. Error bars represent the standard error of the mean of three experiments carried out in triplicate. (**b**) Effect of MPO inhibitor on cell viability was determined by treating HeLa cells with increasing concentrations of 4-Aminobenzoic hydrazide with either 0 mM or 50 mM NaCl. After 24 and 48 hours viability was determined and normalized to untreated cells (0 mM NaCl). Error bars represent the standard error of the mean of three experiments carried out in triplicate. **p < 0.01 when compared to 0 μM 4ABAH. NaCl (mM) values are over and above that found in DMEM (110 mM).

Since neutrophils use the enzyme myeloperoxidase (MPO) to convert Cl^−^ to HOCl within phagosomes^10^, and intracellular HOCl production is known to occur in the gut epithelium of fruit flies^9^, and we had also detected HOCl production in HeLa cells, we investigated the effect of inhibiting MPO in HeLa cells. HeLa cells were infected with eGFP-HSV-1 and cultured in media alone or 50 mM NaCl in the presence of increasing concentrations of 4-Aminobenzoic Hydrazide (4ABAH), a known myeloperoxidase inhibitor which also inhibits HOCl production^10,11^. eGFP-HSV-1 is inhibited in the presence of 50 mM NaCl and the absence of 4ABAH. Viral inhibition is significantly reversed with increasing concentrations of 4ABAH which prevents the conversion of Cl^−^ to HOCl (p = 0.005, Fig. 8a). The combination of NaCl and 4ABAH was not cytotoxic to HeLa cells (viability >70%) (Fig. 8b). This phenotype is similar to that seen in the presence of NPPB, the chloride channel inhibitor. Since MPO is used to convert Cl^−^ to HOCl within phagolysosomes, these results together point to the importance of chloride ion for the antiviral effect and confirm our hypothesis that non-myeloid cells utilize available Cl^−^ to produce HOCl.

## Discussion

Our data suggests that non-myeloid cells have an innate immune mechanism with which they can resist viral infections. This mechanism is dependent on conversion of available chloride ions into HOCl, mediated by a peroxidase. Since HOCl is the active ingredient in bleach which has a broad antiviral range against DNA, RNA, enveloped and non-enveloped viruses, the antiviral mechanism should work against different types of viruses. Our *in-vitro* data confirm this. However, the exact mechanism by which HOCl inhibits viruses within the cell needs to be investigated.

HOCl production in phagocytic cells such as neutrophils and macrophages requires MPO, which is known to be expressed at high levels in these cells from microarray-based trancriptomic studies (http://biogps.org/)^12^. More recent RNAseq (GTEx database) (https://www.gtexportal.org/home/) and SAGE (serial analysis of gene expression) (https://cgap.nci.nih.gov/) studies, however, have shown that MPO is also expressed at lower levels in a much broader spectrum of cells, including those from lung and skin tissue (http://www.proteinatlas.org/ENSG00000005381-MPO/tissue). Low level expression of MPO in epithelial cells might thus be an explanation for the results observed in this study.

Another possibility is that other enzymes could have a role similar to that of MPO. NADPH oxidase (Nox) and dual oxidase (Duox) are both members of the Nox and Duox family enzymes^13^. Five Nox and two Duox enzymes have been identified in humans, compared to one Nox and one Duox in *Drosophila*. Nox help in the production of superoxide anion. Duox have both the NADPH oxidase domain along with an extracellular peroxidase homology domain, a transmembrane domain, and a calcium modulated EF hand domain. Due to the presence of the peroxidase domain, Duox help convert H_2_O_2_ to HOCl in the presence of Cl^−^ in *Drosophila* and are thought to convert H_2_O_2_ to hypothiocyanate (OSCN^-^) in the presence of thiocyanate anion (SCN^-^) in mucosal epithelium of mammals^13^. However in both these models, since Duox is only thought to be present on the apical membrane, the reactive oxygen species (ROS) produced is thought to be extracellular (i.e. into the fluid lining the gut or the respiratory tract) as demonstrated in 3D culture model of normal human bronchial epithelial (NHBE) cells infected with influenza virus A (H1N1 and H3N2)^14^. Whist there was recovery of barrier integrity in NHBE cells after H1N1 infection, infection with H3N2 was associated with loss of barrier integrity and enhanced cell death^14^. Suppression of H_2_O_2_ by catalase or GKT136901 led to increased viral RNA expression and viral particle release^14^. Both H1N1 and H3N2 influenza A viruses down regulated Duox 1 and dual oxidase maturation factor A1 (Duox A1). However Duox 2 and dual oxidase maturation factor A 2 (Duox A2) mRNA expression was significantly increased after H1N1 infection (which was less pathogenic in this model) and less so with H3N2 suggesting the conversion of H_2_O_2_ to HOCl possibly reduced the pathogenicity of the H1N1 infection^14^. Infection with rhinovirus has also been reported to lead to an increase in Duox 2 expression^15^.

The evidence presented above suggests that Duox-mediated increased HOCl activity might lead to an inhibition of viruses outside of the cell. In our experiments increased fluorescence was seen after 30-minute incubation with HCSe/R19-S dyes. Since any excess dye was washed away before fluorescence was measured, it is likely that the HOCl detected in our experiments in intracellular. In *Drosophila*, intracellular HOCl production in gut epithelial cells has been reported in response to ingestion of a bacterial lysate^9^. The authors also reported that a Duox knock down mutant was incapable of producing intracellular HOCl^9^. Strengert *et al*. have also shown that Duox 1/2/A1/A2 are all expressed in the nuclear membrane of normal human bronchial epithelial cells suggesting intracellular HOCl production is possible within epithelial cells^14^. It has also been reported that the peroxidase component in Duox 2 is a heme peroxidase which is inhibited in the presence of sodium azide^14^. We used 4ABAH, a known myeloperoxidase inhibitor; and myeloperoxide is also a heme peroxidase^16,17^. Whether 4ABAH also inhibits the heme peroxidase found in Duox 2 remains to be verified. There are also other mechanisms by which cells can produce HOCl. For example sea-urchins can produce HOCl from H_2_O_2_ and NaCl in the presence of copper ions independent of MPO^18^.

It is thought that viruses and their host cells co-evolved over millions of years^19^. Recently, 214 vertebrate-associated RNA viruses have been identified in fish, amphibians and reptiles adding to the body of evidence^20,21^. If co-evolution is true, it stands to reason that cells would have evolved to fight viral infections at an early stage (i.e. when life began in the marine environment), and potentially used the most commonly available resource – chlorine^22^. White leg shrimp (*Litopenaeus vannamei)*, a euryhaline shrimp that can adapt and survive in a wide range of salinities, is known to get infected with white spot syndrome virus. Outbreaks of white spot syndrome virus are common in shrimp farms during the rainy season, with a possible association with lower salinity and temperatures. It has recently been reported that shrimp are more susceptible to white spot syndrome virus when the water is at a lower salinity, suggesting that chloride ions may have an antiviral effect in marine organisms as well^23^. This potential relationship between virus, eukaryotic co-evolution and antiviral mechanism is interesting and needs further research.

Recent evidence may also support our hypothesis that HOCl has an important role to play in epithelial defense. Women with the GG polymorphism in promoter region of the myeloperoxidase gene G463A have been reported to produce more myeloperoxidase and have a lower possibility of developing cervical cancer than those who have the heterozygous genotype GA^24^. Since high risk human papilloma viruses (HPV) cause cervical carcinoma, it is possible that the inability to produce HOCl (either by phagocytic/epithelial cells) and thereby effectively clear HPV infections in individuals with the GA haplotype could play a causative role.

Jantsch *et al*. report that activation of macrophages in the presence of high NaCl concentrations led to increased nitric oxide production within macrophages helping eliminate bacterial and parasitic skin infections^25,26^. A high salt diet led to increased Na^+^ accumulation in the skin thereby boosting macrophage activation which helped clear bacterial infections, pointing to the important role NaCl plays in host immunity^25^.

In parallel with this study, we conducted a clinical trial of hypertonic saline nasal irrigation and gargling for upper respiratory tract infections which showed a significant reduction in the duration of illness in the intervention arm and control arm along with a reduction in viral shedding (manuscript submitted). Hence both sets of data support our view that non-myeloid cells make use of a source of chloride ions to produce HOCl which helps to fight viral infections. Our findings open the field to a new avenue of therapy based on harnessing this host immune defense mechanism. Supplying NaCl locally could also help treat other common illnesses such as the cold sores, genital herpes, and viral gastroenteritis. Upper respiratory tract infections could be cured before they become lower respiratory tract infections or cause exacerbations in those with asthma, chronic obstructive pulmonary disease, cystic fibrosis, etc. Even more exciting is the possibility of using this simple intervention as a prophylactic tool.

## Methods

### Virus replication assays

#### HSV

HeLa cells (purchased from European Collection of Authenticated Cell Cultures) were seeded in black clear-bottomed 96-well plates (1 × 10^4^ cells/well) in 100 µl Dulbecco’s modified eagle medium (DMEM)/5% foetal calf serum (FCS)/1% penicillin-streptomycin (PS) and incubated overnight at 37 °C for cells to adhere. The next day, media was removed, and the cells infected with 25 µl HSV-1-eGFP at multiplicity of infection (MOI) 0.5. After adsorption for 1 hour at 37 °C, inoculum was removed and replaced with increasing concentrations (0, 10, 20, 30, 40, 50 and 100 mM) of NaCl in phenol red-free medium. Virus replication was monitored as a function of eGFP fluorescence over multiple rounds of virus replication (using POLARstar OPTIMA plate reader (BMG Labtech) with excitation wavelength of 490 nm and emission wavelength of 520 nm). The slopes of replication over the linear growth phase were calculated and normalised to untreated (0 mM NaCl) cells.

#### Effect of NaCl on the virus

HSV-1-eGFP was pre-incubated with increasing concentrations of NaCl for 0, 1 or 2 hours before infecting HeLa cells (MOI 0.5). After adsorption, the inoculum was removed and replaced with media and cultured as above.

#### Antiviral effect when NaCl is present during viral adsorption and/or replication

HeLa cells were infected with HSV-1-eGFP at MOI 0.5 in the presence or absence of increasing concentrations of NaCl. After adsorption for an hour, the inoculum was removed and replaced with the appropriate concentration of NaCl or medium and cultured as given above.

#### MHV68

3T3 cells (purchased from American Type Culture Collection) were seeded in black clear-bottomed 96-well plates (1 × 10^4^ cells/well) in 100 µl DMEM/5% calf serum/1% PS and incubated overnight at 37 °C for cells to adhere. The next day, media was removed, and the cells infected with 25 µl MHV68-eGFP at MOI 0.015. After adsorption for 1 hour at 37 °C, inoculum was removed and replaced with increasing concentrations (0, 10, 20, 30, 40, 50 and 100 mM) of NaCl in phenol red-free medium. Virus replication was monitored as a function of eGFP fluorescence over multiple rounds of virus replication (using POLARstar OPTIMA plate reader (BMG Labtech) with excitation wavelength of 490 nm and emission wavelength of 520 nm). The slopes of replication over the linear growth phase were calculated and normalised to untreated (0 mM NaCl) cells.

#### RSV

HeLa cells were seeded in black clear-bottomed 96-well plates at 1 × 10^4^ cells/well in 100 µl DMEM/5% FCS/1% PS and incubated overnight at 37 °C for cells to adhere. The next day, media was removed, and cells infected with 25 µl RSV-eGFP at MOI 0.5 diluted in media or in NaCl in increasing concentrations (0, 10, 20, 30, 40 and 50 mM). After incubation for 1 hour at 37 °C, inoculum was removed and replaced with the appropriate concentration of NaCl in phenol red free medium or phenol red free media alone and fluorescence was measured (as above). The slopes of replication over the linear growth phase were calculated and normalised to untreated (0 mM NaCl) cells.

#### Influenza A virus

A549 cells (gratefully received from Professor Richard Randall, School of Biology, University of St. Andrews, UK) were seeded in clear 24-well plates (1 × 10^5^ cells/100 µl/well) in DMEM/10% FCS/1%PS and incubated at 37 °C. After 24 hours, the cells were washed twice with 1 ml PBS before cells were infected with IAV diluted to MOI 0.01 in serum-free DMEM with 1 μg/ml trypsin. After adsorption for an hour at 37 °C, the inoculum was replaced with increasing concentrations (0, 10, 20, 30, 40, 50 and 100 mM) of NaCl made in IAV infection media. After 12 hours, cells were washed with 1 ml PBS and 200 μl Trizol reagent was added into each well. The contents were transferred to a 1.5 ml Eppendorf tube, 40 μl chloroform was added to each tube and incubated at room temperature for 2–3 min, then centrifuged for 15 min at 12000 g at 4 °C. The upper aqueous phase (whose volume = V) was collected in a clean 1.5 ml Eppendorf tube. A mixture of 2.5 V 75% ethanol, 0.1 V 3 M sodium acetate (pH 5.2) and 4 μl glycogen (5 mg/ml) was added and the mixture kept at −80 °C for more than 2 hours to precipitate RNA. RNA was pelleted at 12000 g for 30 min at 4 °C and the supernatant was removed. The RNA pellet was washed twice by adding 500 μl 75% ethanol and centrifuging for 10 min at 12000 g at 4 °C. The pellet was dried and dissolved in 50 μl water. RNA concentration and quality was measured by Nanodrop spectrophotometer (Thermo). A quantitative RT-PCR was performed to quantify IAV nucleoprotein mRNA using the Verso 1-step RT-qPCR Mix, low ROX kit (Thermo Fisher), according to manufacturer’s instruction. The Master Mix, containing 5 μl 2 × 1-Step qPCR Low ROX Mix, 0.1 μl Verso Enzyme Mix, 0.5 μl RT enhancer, 0.8 μl primer and 0.1 μl probe was added. Primers targeted against the NP region and probe were (Forward 5′-gtgcaaagaaacctcccatt-3′, Reverse 5′-gccctcatgtctgaggttct-3′, Probe: Universal probe library: UPL#48). The housekeeping gene hypoxanthine-guanine phosphoribosyl transferase (HPRT) 1 gene was the quantitative calibrator. Then 3.5 μl of RNA (containing 20 ng RNA) was added and RT-PCR performed. The cycle threshold (Ct) values of NP gene was normalised to that of the HPRT gene. Then NP gene quantities were normalised to that in the absence of NaCl. Cell viability was determined after 24 and 48 hours by addition of 10 µl CellTiter Blue (Promega).

### CV-B3

HuH-7.5 cells (gratefully received from Prof. Nicole Stonehouse, University of Leeds) were seeded onto a black 96-well plate at 2 × 10^4^ cells/well in 100 μl of phenol red-free DMEM/5% FCS/1% PS and incubated overnight at 37^ο^C in 5% CO2. CV-B3 stock was diluted 1 in 5 with phenol red-free DMEM, and 25 μl of diluted virus was added to each well with 25 μl of phenol red-free DMEM added to the control wells. The virus was adsorbed to the cells for 1 hour at 37 °C. After 1 hour, virus and media were removed from the plate and replaced with 100 μl of varying concentrations of NaCl. The NaCl dilutions were prepared in a serial dilution series at concentrations of 0, 10, 20, 30, 40, 50 and 100 mM, diluted in phenol red-free DMEM. Replication was monitored as a function of eGFP fluorescence using a POLARstar OPTIMA plate reader, between 3–33 hours post-infection. Plates were incubated at 37 °C in a humidified incubator with 5% CO2 between plate reads.

### HCoV-229E

Huh-7 cells were seeded in black clear-bottomed 96 well plates at 2 × 10^4^ cells/well with 100 µl of DMEM/10% FCS/1%PS and incubated overnight at 37 °C for cells to adhere. The following day, media was removed, and the cells were infected with HCoV-229E-GFP in 30 µl phenol red free, serum-free DMEM to obtain an MOI of 0.05. After adsorption for 1 hour at 37 °C, increasing concentrations of NaCl in phenol red-free DMEM with 10% FCS were added to a final concentration of 0, 10, 20, 30, 40, 50, and 100 mM of NaCl in addition to that in DMEM with 5% FCS. Plates were incubated at 33 °C. Virus replication was measured hourly between 24 and 48 hours as a function of eGFP fluorescence. A POLARstar OPTIMA plate reader (BMG Labtech) was used for replicates 1 and 2. A CLARIOstar OPTIMA (BMG Labtech) with the addition of 5% CO2 was used for replicate 3. An excitation wavelength of 490 nm and emission wavelength of 520 nm were used. The slopes of replication over the linear growth phase were calculated and normalised to replication in cells treated with no additional NaCl.

### Cell Viability assays

Cells were seeded in clear 96-well plates as described above. Cells were then exposed in multiple wells to identical conditions to the uninfected control cells in the corresponding virus replication assay.

Two hours before the allocated time (for e.g. 22 and 46 hours post-treatment with NaCl), 10 µl/well of CellTiter Blue reagent (CTB; Promega; a dye which produces a fluorescent signal relative to the number of viable cells present) was added to each set of duplicates for 24 hours and 48 hours cell viability, respectively. These were then incubated for 2 hour at 37 °C in a humidified incubator with 5% CO2 before fluorescence measurement (POLARstar OPTIMA plate reader). Plates were incubated at 37 °C in a humidified incubator with 5% CO2 between plate reads. Cell viability was normalised to untreated cells (0 mM NaCl). Viability below 70% was used as a threshold for cytotoxicity^27^.

### Quantification of HSV-1 by plaque assay

2 × 10^4^ HeLa cells were seeded in 96-well plates before infecting with HSV-1 (MOI 0.5) and harvesting supernatant at 6, 12, 24, 36, or 48 hours p.i. For virus quantification, 1 × 10^5^ cells Vero cells were seeded in 24-well plates before infecting with 150 μl virus supernatant for 1 hour at 37 °C. Inoculum was removed and cells overlaid with 1 ml DMEM/5% FCS with 0.8% agarose before fixing with 1% formaldehyde, removing agarose plugs and staining cells with crystal violet after 72 hours. Plaques were counted, and titers calculated as PFU per ml.

### Chemical inhibitor experiments

HeLa cells were seeded in black clear-bottomed 96-well plates at 1 × 10^4^ cells/well in 100 µl DMEM/5% FCS/1% PS and incubated overnight at 37 °C for cells to adhere. The following day the medium was removed, and cells were pre-treated with increasing concentrations of sodium/chloride channel inhibitor (ralfinamide (Tocris) − voltage gated sodium channels; benzyl amiloride (Sigma) − epithelial sodium channels; NPPB (Sigma) − chloride channels) or the myeloperoxidase inhibitor 4-Aminobenzoic hydrazide (0, 1, 2.5, 5, 10, 20 or 40 μM) (Abcam). The following day the inhibitor was removed, and cells were infected with HSV-1-eGFP at a MOI of 0.5. After incubation for 1 hour at 37 °C, the inoculum was removed and replaced with the appropriate concentration of channel blocker or the myeloperoxidase inhibitor 4-Aminobenzoic hydrazide (4ABAH) and 0 or 50 mM of NaCl. Virus replication was monitored as a function of eGFP fluorescence over multiple rounds of virus replication. The slopes of replication over the linear growth phase were calculated and normalised to untreated cells.

To determine if there were any cytotoxic effects due to the inhibitors, HeLa cells were pre-treated with increasing concentrations of inhibitor for 24 hours before mock-infecting with media for 1 hour. Inoculum was removed and replaced with increasing concentrations of corresponding channel inhibitor and either 0 mM or 50 mM NaCl and cell viability assayed at 48 hours as described above.

### HOCl production in HeLa cells

HeLa cells were seeded in black clear-bottomed 96 well plates at 1.5 × 10^4^ cells/well in 100 µl DMEM/5% FCS/1% PS and incubated overnight at 37 °C for cells to adhere. The next day, media was removed and cells infected with 25 µl media or HSV-1 SC16 110 lacZ^28^ at MOI 0.5. After incubation for 1 hour at 37 °C, inoculum was removed and replaced with 0, 25, 50, or 100 mM NaCl in phenol red-free medium. At 6 hours post-infection, cells were washed with 100 μl PBS, and 50 μl of 10 μM HCSe^8^ (kindly provided by S. Wu) or R19-S^9^ (Futurechem) solution was added per well. After 30 minutes at room temperature in the dark, wells were washed twice with 100 μl PBS and 50 μl of PBS added per well before fluorescence measured (excitation wavelength of 510 nm and emission wavelength of 542 nm). Fluorescence values were normalized to uninfected cells in the presence of corresponding concentrations of NaCl.

### Statistical analysis

Where appropriate, p values were calculated using unpaired two-tailed t-test for unequal variances. p values < 0.05 are reported.

## Data Availability

The data generated in the study are available from the corresponding author on reasonable request.
